# *Rickettsia parkeri* and *Candidatus* Rickettsia andeanae in Tick of the *Amblyomma maculatum* Group, Mexico

**DOI:** 10.3201/eid2504.181507

**Published:** 2019-04

**Authors:** Jesús Delgado-de la Mora, Sokani Sánchez-Montes, Jesús D. Licona-Enríquez, David Delgado-de la Mora, Christopher D. Paddock, Lorenza Beati, Pablo Colunga-Salas, Carmen Guzmán-Cornejo, Maria L. Zambrano, Sandor E. Karpathy, Andrés M. López-Pérez, Gerardo Álvarez-Hernández

**Affiliations:** Instituto Nacional de Ciencias Médicas y Nutrición Salvador Zubirán, Mexico City, Mexico (J. Delgado-de la Mora);; Universidad Nacional Autónoma de México, Mexico City (S. Sánchez-Montes, P. Colunga-Salas, C. Guzmán-Cornejo, A.M. López-Pérez);; Centro Médico Nacional Siglo XXI, Mexico City (J.D. Licona-Enríquez);; Instituto Tecnológico de Sonora, Sonora, Mexico (D. Delgado-de la Mora);; Centers for Disease Control and Prevention, Atlanta, Georgia, USA (C.D. Paddock, M.L. Zambrano, S.E. Karpathy);; Georgia Southern University, Statesboro, Georgia, USA (L. Beati);; Universidad de Sonora, Sonora (G. Álvarez-Hernández)

**Keywords:** Rickettsia parkeri, spotted fever group rickettsiae, Mexico, vector-borne infections, ticks, bacteria, Candidatus Rickettsia andeanae, rickettsia

## Abstract

We report *Rickettsia parkeri* and *Candidatus* Rickettsia andeanae in ticks of the *Amblyomma maculatum* group collected from dogs in Sonora, Mexico. Molecular characterization of these bacteria was accomplished by DNA amplification and sequence analysis of portions of the rickettsial genes *gltA*, *htrA*, *ompA*, and *ompB*.

*Rickettsia parkeri*, a member of the spotted fever group *Rickettsia* (SFGR), was initially identified in *Amblyomma maculatum* ticks in 1937, but not until 2004 was the first confirmed human infection reported (*1*). Through 2018, at least 9 cases of *R. parkeri* rickettsiosis have been identified across several mountainous locations in southern Arizona close to the US–Mexico border, and ticks of the *A. maculatum* group infected with *R. parkeri* have been collected from this region (*2*).

Epidemic levels of Rocky Mountain spotted fever (RMSF), a severe and frequently fatal spotted fever rickettsiosis, have been identified throughout regions of northern Mexico, including the state of Sonora (*3*). *R. rickettsii* (the agent of RMSF) is the only SFGR species linked with spotted fever rickettsiosis in Sonora; nonetheless, a unique lineage of *R. parkeri* associated with the *Dermacentor parumapertus* tick was reported recently from northern Mexico (*4*). Sonora shares an international border with Arizona; the recent identification of *R. parkeri* in *Amblyomma* ticks in southern Arizona (*2*) prompted our investigation during July 2016 for ticks in northern Sonora to determine whether another pathogenic SFGR species could also exist in Mexico.

The study area comprised 3 localities in Sonora (Yecora, 28°22′16″N, 108°55′32″W; Matarachi, 28°73′54′′N, 108°95′87′′W; and Mulatos, 28°65′85′′N, 108°74′78′′W) (Figure), each with a humid subtropical climate situated at ≈1,500 m, with vegetation comprising predominantly oak forests and grasslands (Appendix Figure, panel A). During August 2016 and September 2017, we inspected free-roaming dogs (*Canis lupus familiaris*) for attached or crawling ticks (Appendix Figure, panel B). We identified ticks using morphological characteristics (*5*).

**Figure:**
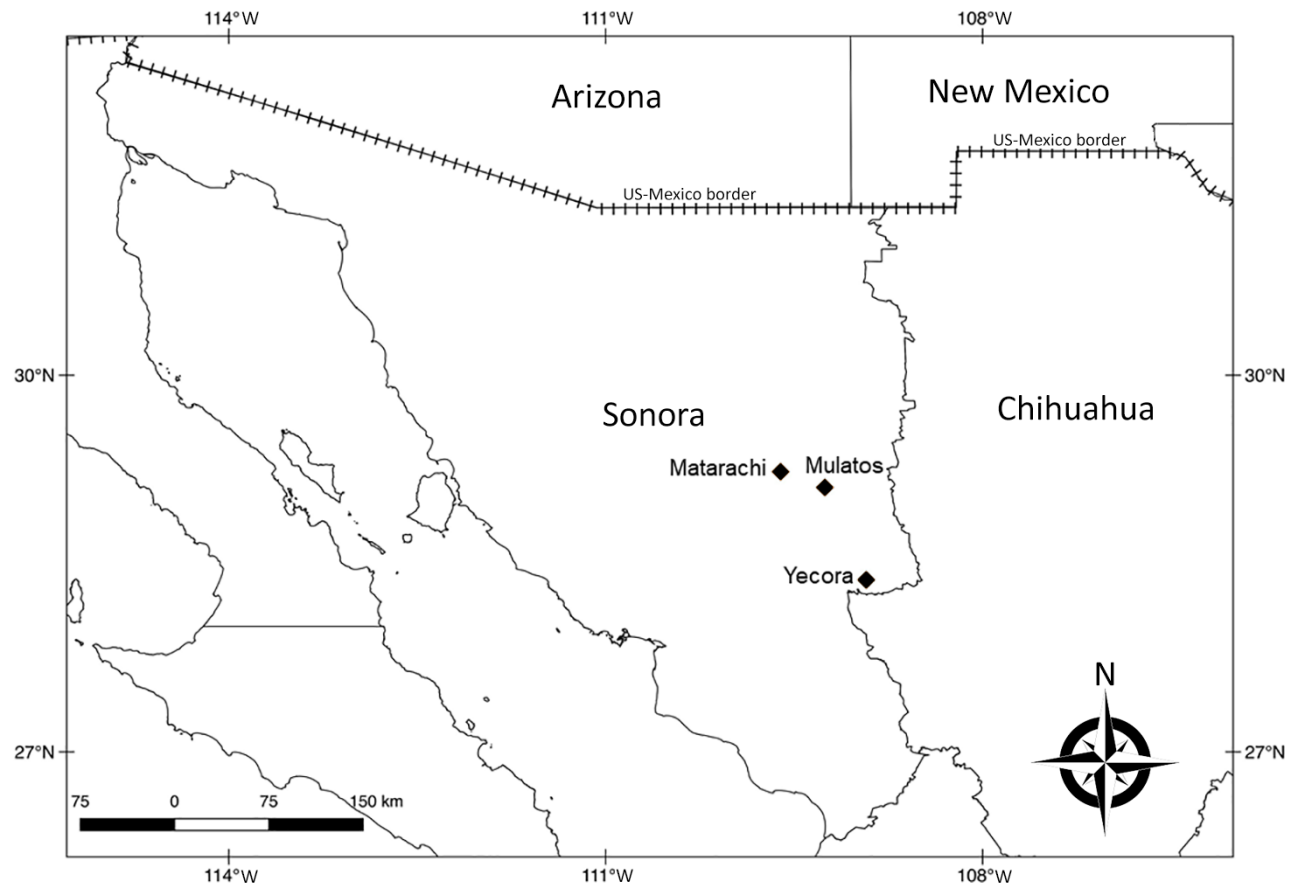
Locations where ticks of the *Amblyomma maculatum* group were collected (diamonds) in a study of *Rickettsia parkeri* and *Candidatus* Rickettsia andeanae, Sonora, Mexico. A layer of Google Maps was used to construct the figure.

For ticks collected in 2016, we dissected a portion of the posterolateral idiosoma from each specimen and extracted DNA using a DNeasy Blood and Tissue Kit (QIAGEN, https://www.qiagen.com). We screened DNA extracts using a *Rickettsia* genus–specific real-time PCR with primers PanR8_F and PanR8_R, probe PanR8_P (*6*), and 5 μL of DNA template. Samples with cycle threshold values <40 were tested by a PCR targeting a segment of the *omp*A gene using primers *Rr*190.70p and 190–701 (*7*). We also tested 1 positive sample using a nested PCR to amplify a segment of the 17-kDa antigen gene with primers R17122 and R17500 (*8*) in the primary amplification and TZ15 and TZ16 (*9*) in the nested amplification. Specimens collected in 2017 were processed similarly; however, we screened DNA extracts using a conventional PCR to amplify a segment of the *gltA* gene (*4*). We subsequently tested positive samples by conventional PCRs targeting fragments of the *ompA* and *ompB*, genes using primers and conditions described elsewhere (*4*). PCR products were sequenced using an ABI 3500 genetic analyzer with a BigDye Terminator v3.1 Cycle Sequencing Kit (Applied Biosystems, https://www.thermofisher.com). Sequences were assembled using Geneious R10 (Biomatters Ltd., https://www.geneious.com) and compared with GenBank data using blastn analysis (http://blast.ncbi.nlm.nih.gov/Blast.cgi).

We collected a total of 31 *A. maculatum* group ticks from northern Sonora (14 specimens from Yecora in 2016 and 17 from Matarachi and Mulatos in 2017). Three ticks collected in 2017 were deposited as voucher specimens in the Colección del Laboratorio de Acarología, Universidad Nacional Autónoma de México (Mexico City, Mexico).

We amplified a sequence demonstrating complete identity to the homologous 590-bp segment of the *omp*A gene of *R. parkeri* strain Maculatum 20 (GenBank accession no. U43802) from 1 (7%) of 14 ticks collected in 2016. We further amplified sequences demonstrating complete identities to the homologous segments of the *ompA* (590 bp) and 17-kDa antigen (208 bp) genes of *Candidatus* Rickettsia andeanae (GenBank accession nos. KF179352 and KY402193, respectively) from another tick in this group and sequences revealing complete identity to each other and to the homologous segments of the *glt*A (760 bp), *omp*B (760 bp), and *omp*A (490 bp) genes of *R. parkeri* strain Portsmouth (GenBank accession no. CP003341.1) from 10 (71%) of 14 ticks evaluated from the 2017 collection. 

We identified DNA of *R. parkeri* and *Candidatus* Rickettsia andeanae in *A. maculatum* group ticks in northern Sonora. *R. parkeri* causes a disease less severe than RMSF and should be suspected in patients with an eschar, rash, and lymphadenopathy (*1*). The results of this investigation suggest that *R. parkeri* could contribute to at least some of the cases of spotted fever rickettsiosis described in Sonora and possibly in other regions of Mexico where *A. maculatum* group ticks are found. Differentiation between these 2 diseases is important, principally because there are no reports of fatal disease caused by *R. parkeri.* Nonetheless, clinical suspicion of any SFGR requires immediate treatment with doxycycline. *Candidatus* Rickettsia andeanae, a SFGR of undetermined pathogenicity, has been detected in the United States and Central and South America (*2**,**10*). Our findings highlight the need for specific diagnostic tests for SFGR in Mexico that can identify other potential SFGR of public health concern in this country.

Appendix Additional information on study of *Rickettsia parkeri* and *Candidatus* Rickettsia andeanae, Sonora, Mexico.
