# Supplementary material for: Rickettsia parkeri and Candidatus Rickettsia andeanae in Tick of the Amblyomma maculatum Group, Mexico
Source: Emerg Infect Dis. 2019 Apr;25(4):836–8. doi: 10.3201/eid2504.181507 (PMC6433039; doi:10.3201/eid2504.181507)
Supplement: Appendix — Additional information on study of Rickettsia parkeri and Candidatus Rickettsia andeanae, Sonora, Mexico. [file 18-1507-Techapp-s1.pdf]

Article DOI: https://doi.org/10.3201/eid2504.181507

Rickettsia parkeri and *Candidatus* Rickettsia andeanae in *Amblyomma maculatum* Ticks, Mexico

Appendix

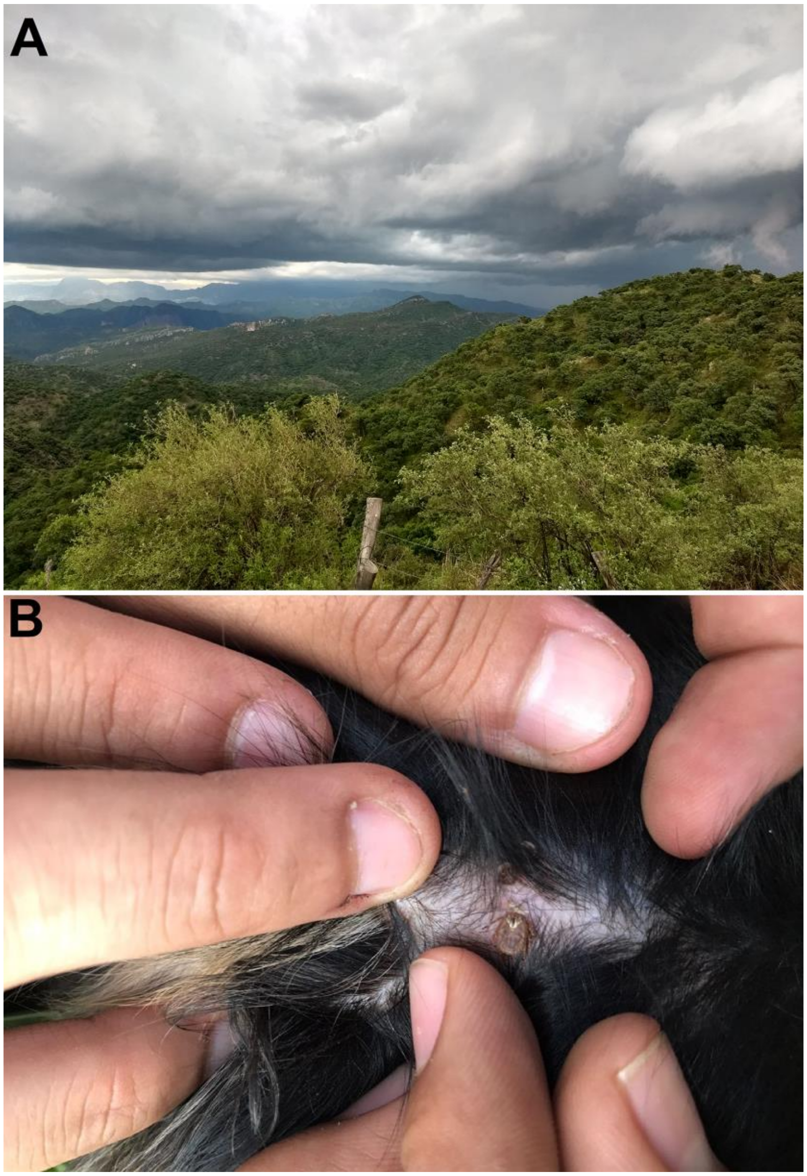

Appendix Figure. A) The areas of study consisted predominantly of oak forest and grasslands. B) *Amblyomma maculatum* tick attached at the skin of a free-roaming dog.
